# Effect of exogenous salicylic acid spray on enhancing cold tolerance in ‘Northland’ blueberry plants under low-temperature stress

**DOI:** 10.1371/journal.pone.0338327

**Published:** 2025-12-10

**Authors:** Xuedong Tang, Jianxin Li, Xue Gao, Binghan Liu, Ruixue Guo, Xiaojia Liu

**Affiliations:** 1 College of Horticulture, Jilin Agricultural University, Changchun, Jilin, China; Bahauddin Zakariya University, PAKISTAN

## Abstract

Blueberry is a small berry with significant economic value; however, in the northern regions of China, low temperatures severely affect blueberry growth during early spring. Salicylic acid (SA), an important phytohormone, plays a crucial role in various physiological and metabolic responses induced by abiotic stress. This study utilized 2-year-old ‘Northland’ blueberry plants as experimental materials to investigate the physiological responses to different concentrations of SA supplementation under low-temperature stress. The results indicated that SA concentration had varying effects on enhancing the cold resistance of blueberry plants under different low-temperature conditions. At a stress temperature of 4°C, a concentration of 2.5 mM SA was most effective in increasing the content of osmoregulatory substances, enhancing the activity of antioxidant system enzymes, reducing reactive oxygen species (ROS) accumulation, up-regulating the ascorbate-glutathione (AsA-GSH) cycle, and elevating the expression level of the *VcICE1* compared to other concentrations. Conversely, under low-temperature stress below 0°C (including 0°C and −4°C), supplementation with 0.5 mM SA resulted in the most significant improvement compared to the control. Therefore, the study findings suggest that the application of 0.5 mM SA enhances the cold tolerance of blueberries. However, additional validation is required to ascertain its efficacy when applied in early spring fields in northern China.

## Introduction

Blueberry (*Vaccinium* ssp.) is a perennial shrub belonging to the genus Vaccinium within the family Ericaceae (*Rhododendron*) [1]. The fruit of the blueberry is rich in flavonoids and polyphenols, which exhibit antiseptic and anti-inflammatory properties [2,3]. Additionally, blueberries contain significant amounts of anthocyanins, which can reduce the generation of free radicals—byproducts of human metabolism—and inhibit the activity of enzymes responsible for cancer cell reproduction [4,5]. Currently, blueberries can be consumed fresh and are also widely utilized in brewing and jam production [5,6]. Consequently, the blueberry industry has experienced rapid growth in recent years, leading to an expansion of cultivated areas [7]. However, in early spring in northern China, the occurrence of inverted spring cold poses a significant threat, as the flower and leaf buds of blueberries have already begun to sprout and are highly susceptible to cold and frost damage. This vulnerability can severely impact yield and economic returns.

SA is a small phenolic compound that naturally occurs in plants and plays crucial physiological roles, including the regulation of plant growth and yield, induction of stomatal closure, heat production, flowering, inhibition of root uptake of inorganic ions such as potassium and phosphorus, promotion of meristematic and adventitious root formation, and overall influence on plant growth [8]. As a significant phytohormone, SA enhances resistance to various abiotic stresses, including drought, cold damage, salt damage, and heavy metal toxicity, across a range of plant species, such as baked tobacco [9], tomato [10], cucumber [8], and wheat [11]. Studies demonstrate that exogenously applied SA at appropriate concentrations enhances plant tolerance to low-temperature stress [12]. This is achieved through modulation of proline biosynthesis, promoting the accumulation of osmoprotective compounds, and enhancing the activity of antioxidant enzymes. These enzymes effectively scavenge low-temperature-induced ROS, thereby reducing membrane lipid peroxidation [13,14]. However, high concentration SA not only abolish this protective effect during low-temperature stress but also exacerbate cellular damage and may induce potential secondary stress [15]. While numerous studies have investigated the effects of SA on plant osmoregulatory substances, cell membrane permeability, antioxidant systems, and the expression of cold-resistant genes in response to low-temperature stress, there is a notable scarcity of research focused on the enhancement of cold resistance in blueberries [16].

The extent of damage to the cell membrane structure is influenced by both the intensity and duration of the stressor, as well as the resistance of the crop variety [17]. Increased damage to a plant correlates with a greater release of substances, resulting in a more conductive medium. Malondialdehyde (MDA), a significant byproduct of membrane lipid peroxidation, serves as an indicator of the degree of cell membrane disruption [18]. Research indicates that MDA levels progressively rise with prolonged exposure to stress; however, after a certain duration, MDA levels begin to decline [19].

Under abiotic stress, the equilibrium between the production and elimination of ROS within the plant is disturbed, leading to an accumulation of ROS. Studies have demonstrated that superoxide anion (O_2_^‧-^) and hydrogen peroxide (H_2_O_2_) play significant roles in signal sensing, transduction, and adaptation to adverse stress in plants [20]. Under stress conditions, plants exercise a direct protective function by stimulating the activity of antioxidant enzymes such as superoxide dismutase (SOD), catalase (CAT), and peroxidase (POD), or by initiating non-enzymatic mechanisms like the AsA-GSH cycle to scavenge excess ROS [21]. The magnitude of these activities is related to plant resistance. The AsA-GSH cycle is a crucial antioxidant system involved in scavenging intracellular ROS during both plant development and stress conditions. Monodehydroascorbate reductase (MDHAR) and dehydroascorbate reductase (DHAR) reduce dehydroascorbic acid to reduced ascorbic acid using NADPH and glutathione (GSH) as electron donors, respectively [22]. Finally, glutathione reductase (GR) reduces oxidized glutathione to GSH. There is a correlation between changes in the activities of ascorbate peroxidase (APX) and GR and plant cold tolerance. Additionally, the contents of all osmoregulatory substances in plants change under stress conditions; among these, the alterations in the levels of soluble proteins (SP), soluble sugars (SS), and free proline (Pro) have been studied most frequently [23]. Relevant research has shown that low-temperature stress can increase the levels of free proline and soluble sugars in plants, thereby alleviating freezing-induced cell dehydration [21,24].

The aim of this study was to investigate the effects of various concentrations of exogenous SA on the cold resistance of blueberry seedlings subjected to low-temperature stress. By measuring the physiological indices of blueberry leaves and the expression levels of cold resistance-related genes *VcICE1* and *VcCBF3*, we elucidated the physiological mechanisms through which SA influences the cold resistance response in blueberry. The optimal concentration of SA was supplemented exogenously to mitigate the damage caused by low-temperature stress during the early spring growth of blueberries. This study provides valuable insights for the cultivation of blueberries in cold regions, facilitating effective prevention of early spring freezing damage to the plants.

## Materials and methods

### Plant growth conditions and treatments

Two-year-old semi-highbush blueberry ‘Northland’ potted plants were utilized as experimental material. Uniformly grown plants were selected and transplanted into nutrient pots measuring 18 cm in diameter, 12 cm at the bottom diameter, and 20 cm in height during the spring months of April and May, with each plant being planted individually. The pots were incubated in a thermostatic chamber in a controlled environment (22 ± 1°C, 16 h photoperiod). The plants were cultivated until their leaves were fully expanded.

Approximately 150 plants exhibiting optimal growth conditions and height were selected for the experiment and sprayed with SA on both the front and back sides of their leaves. The SA treatment concentrations were categorized into six groups: 0 (CK), 0.5 (A1), 1.0 (A2), 1.5 (A3), 2.0 (A4), and 2.5 (A5) mmol·L ⁻ ¹. Each treatment consisted of 24 plants, with three replications of eight plants per replication. The spray volume was 0.5 L per plant, with 1 m between each treatment group. Spraying occurred at 72 h, 48 h, and 24 h prior to the low-temperature treatment, with a control temperature of 25°C. The low-temperature treatment involved three different temperatures: 4°C, 0°C, and −4°C. The light intensity of the light incubator (PGXD-450, Shanghai Baidian, China) is 12000 Lux, the humidity is 60%, and the temperature uniformity is ≤± 0.5 °C. The cooling process was initiated by gradually reducing the temperature from room temperature to the designated levels at a rate of 4°C per hour, followed by a stabilization period of 16 h at the set temperatures. A 16-h low-temperature treatment represents the minimum effective dose that will not cause irreversible frostbite. Subsequently, the young leaves at the top of the plants were harvested for the assessment of various physiological indices.

### Determination of SP, SS and free proline

The content of SS was determined using anthrone colorimetric method [25]. The sugars react with anthrone to produce colored compounds, and the absorbance value is measured at 630 nm. Subsequently, the soluble sugar content is calculated based on a working curve. Daily baseline correction was performed once against the solvent at the beginning of each ultraviolet spectrophotometer (L5S, Shanghai Instrumentation & Electronics Group, China) run. Sample absorbance was recorded in triplicate using the same matched pair of 1 cm quartz cuvettes; the maximum acceptable difference among replicates was ≤ 1%.

The SP content was determined using the staining method with Coomassie Brilliant Blue (G-250) [25]. A volume of 0.1 mL of the extract was mixed with 5 mL of G-250 and allowed to react for 2 min. Subsequently, the optical density at 595 nm was measured, and the soluble protein content was calculated based on the established working curve.

The Pro content was determined using the ninhydrin colorimetric method [25]. Toluene was added to the prepared solution, which was then shaken at a constant speed to extract the red substance. After allowing the solution to stratify, the toluene layer was aspirated for colorimetry at a wavelength of 520 nm, and the Pro content was calculated based on the standard curve.

### Determination of chlorophyll content

The determination of chlorophyll a (Chl a) and chlorophyll b (Chl b) content was performed using the ethanol-acetone complex immersion method. A total of 0.2 g fresh leaves were immersed in the extraction solution composed of 80% acetone, anhydrous ethanol, and distilled water in a ratio of 4.5:4.5:1. The samples were treated in the dark for 24 h, after which the OD_470_, OD_645_, and OD_663_ were measured using a spectrophotometer. Chl a = 12.72A_663_ − 2.59A_645_, Chl b = 22.88A_645_ − 4.67A_663_, Total Chl = chlorophyll a + b.

### Determination of relative electrical conductivity (REC) and MDA content

The conductivity measurements were conducted following the method by Li. JX [26], utilizing a DDS-307A conductivity instrument (Shanghai Instrumentation & Electronics Group, China). The initial conductivity of the leachate was recorded as E. Subsequently, the leachate was placed in a water bath and boiled for 30 min, after which it was removed and allowed to cool to room temperature. The conductivity was then measured again, recorded as E0. REC = E/E0 × 100%.

Additionally, the content of MDA was determined using the thio-barbituric aci method [26], with absorbance values measured at wavelengths of A532, A600, and A450, respectively.

### Determination of O_2_^‧-^ and H_2_O_2_

The rate of O_2_^‧-^ production was determined using the hydroxylamine oxidation method [25]. First, 0.5 mL of crude enzyme extract was mixed with 0.5 mL of 50 mmol‧L^-1^ phosphate buffer and 1 mL of 1 mmol‧L^-1^ hydroxylamine hydrochloride. The mixture was shaken thoroughly and allowed to stand at 25°C for 1 h. Subsequently, 1 mL of 17 mmol‧L^-1^ p-aminobenzene sulfonic acid and 1 mL of 7 mmol‧L^-1^ α-naphthylamine were added, and the solution was allowed to stand at 25°C for an additional 20 min before measuring the optical density at 530 nm.

The H_2_O_2_ content was determined using the titanium sulfate precipitation method [25]. One milliliter of the extract was mixed with 0.1 mL of 5% (W/V) TiSO_4_ and 0.2 mL of concentrated ammonia, resulting in the formation of a precipitate (peroxide-Ti complex). The mixture was then centrifuged for 10 min at 10,000 r‧min^-1^. The precipitate was washed three times with acetone and subsequently dissolved in 3 mL of 2 mol‧L^-1^ H_2_SO_4_. To ensure complete dissolution, an additional 7 mL of distilled water was added. Finally, the absorbance was measured at 415 nm.

### Determination of enzyme activities of antioxidant systems

The POD was determined using the guaiacol method at a wavelength of 470 nm. SOD activity was measured via the nitrotetrazolium blue chloride (NBT) photoreduction method at a wavelength of 420 nm. CAT activity was assessed with a UV spectrophotometer, measuring absorbance at 240 nm. APX activity was also measured using a UV spectrophotometer, with absorbance values recorded at a wavelength of 290 nm. The content of GSH was determined by the DTNB colorimetric method, measuring absorbance at 412 nm. The content of AsA was quantified using the bipyridine colorimetric method, with absorbance measured at 534 nm following the color reaction.

### DNA extraction, RNA isolation and qRT-PCR for gene expression analysis

Total DNA from plants was extracted using the CTAB method [26]. Plant RNA was isolated utilizing the Plant RNA Simple Total RNA Kit. cDNA was synthesized with the PrimeScript™ 1st Strand cDNA Synthesis Kit. For gene quantification and relative real-time expression analysis, a 2 × SYBR real-time PCR premix was employed. The real-time quantitative polymerase chain reaction (qRT-PCR) was conducted using *VcGapdh* as the reference gene [25,27]. The sequences of the primer are detailed in Table 1. The results of the qRT-PCR were analyzed using the 2 ^-ΔΔCT^ method to determine relative gene expression.

**Table 1:** Sequences of the primers.

Primer name	Sequence ((5’ → 3’))
*VcICE1-F*	CCCCTTTGACACTGGCTTTG
*VcICE1-R*	CAGACATCGGGAGCAAATGG
*VcCBF3-F*	AGTGAGGAGGAGGAACAACG
*VcCBF3-R*	GACTCACTTTTCAGCGCCAA
*VcGapdh-F*	TCTGCCCCAAGTAAGGAT
*VcGapdh-R*	TGGAGACAATGTGAAGATCG

### Statistical analysis

Data were analyzed using Origin 2021 software (OriginLab Corporation, USA). Statistical analyses were performed using one-way analysis of variance (ANOVA) implemented in SPSS 22.0 (IBM, New York, USA). Principal component analysis (PCA) analysis was also performed with the SPSS 22.0. The treatment group served as the independent variable, while each physiological parameter was treated as a dependent variable. To assess statistical significance, multiple comparisons were conducted using least significant differences (LSD) with a significance level set at *P* < 0.05.

## Results

### Effect of different concentrations of SA on Chl content in blueberry leaves under low temperature conditions

As shown in Table 2, the total chl content was generally lower than that of the CK under normal conditions with SA supplementation, but this difference was not statistically significant. These results suggest that the application of SA under normal conditions had a minimal impact on the photosynthetic activity of the plants.

**Table 2:** Chlorophyll a (Chl a), Chl b and total Chl content of blueberry leaves under low temperature stress at different SA supplementation concentrations.

Treatment		Chl a (mg·g^-1^·FW)	Chl b (mg·g^-1^·FW)	Total Chl content (mg·g^-1^·FW)
25°C	CK	418.97 ± 12.86a	18.88 ± 5.91c	437.85 ± 18.76b
	A1	354.65 ± 28.44c	75.39 ± 8.80a	430.04 ± 35.03ab
	A2	366.36 ± 10.13b	73.99 ± 4.45a	440.35 ± 14.57b
	A3	323.67 ± 31.92d	64.34 ± 9.72a	388.02 ± 41.10c
	A4	330.13 ± 11.16c	64.40 ± 1.44a	394.54 ± 12.59ab
	A5	337.31 ± 20.57c	45.57 ± 4.55b	382.88 ± 16.48c
4°C	CK	294.67 ± 10.90bc	59.53 ± 8.26ab	354.21 ± 16.90b
	A1	305.45 ± 8.96ab	58.84 ± 11.77ab	364.29 ± 19.82b
	A2	275.15 ± 5.31c	53.39 ± 1.81b	328.55 ± 6.59c
	A3	293.96 ± 29.25bc	65.43 ± 7.05ab	359.40 ± 36.23b
	A4	315.47 ± 6.86ab	70.11 ± 2.19a	385.59 ± 8.88a
	A5	323.75 ± 14.11a	61.57 ± 8.34ab	385.32 ± 16.16a
0°C	CK	253.46 ± 5.87b	70.08 ± 7.68b	323.55 ± 11.90b
	A1	272.61 ± 15.07a	85.75 ± 5.89a	358.37 ± 9.41a
	A2	254.58 ± 7.32b	80.44 ± 4.83ab	335.02 ± 9.61b
	A3	213.38 ± 4.34d	56.62 ± 7.33c	270.01 ± 3.13d
	A4	226.97 ± 7.38 cd	53.88 ± 2.15c	280.86 ± 6.06d
	A5	231.52 ± 2.51c	69.52 ± 9.70b	301.05 ± 11.35c
−4°C	CK	206.22 ± 5.31b	63.66 ± 2.16c	269.89 ± 3.25ab
	A1	224.16 ± 8.43a	62.35 ± 0.97c	286.51 ± 7.56a
	A2	214.93 ± 5.40ab	64.94 ± 2.26c	279.88 ± 6.73a
	A3	169.71 ± 13.35c	87.06 ± 6.55a	256.77 ± 19.89b
	A4	201.62 ± 3.03b	74.96 ± 8.97b	276.59 ± 6.76ab
	A5	205.05 ± 13.83b	68.07 ± 4.29bc	273.13 ± 11.31ab

Chl a, chlorophyll a; Chl b, chlorophyll b. The SA treatment concentrations were categorized into six groups: 0 (CK), 0.5 (A1), 1.0 (A2), 1.5 (A3), 2.0 (A4), and 2.5 (A5) mmol·L ⁻ ¹. Significant difference analyses were conducted using one-way ANOVA, with different letters indicating statistically significant differences (LSD, *P* < 0.05).

Supplementation of SA at 4°C stress condition resulted in higher chl a and b content compared to the CK in all groups, except for the A2. In this case, the A4 and A5 exhibited significant differences from CK. However, the total chl content decreased with SA supplementation under 4°C condition when compared to normal temperature. These results suggest that all concentrations of SA treatments, except for the 1.0 mmol‧L^-1^ SA treatment (A2), effectively maintained the photosynthetic capacity of the cells by slowing the degradation rate of chlorophyll.

Supplementation of SA at 0°C condition, resulted in higher chlorophyll content in the A1 and A2 compared to the CK, with increases of 10.76% and 3.55%, respectively. Notably, A1 exhibited a significant difference from CK, while the other treatments were lower than the control. A general trend of decreased chlorophyll content was observed with SA supplementation under 0°C stress when compared to normal temperature, with reductions of 26.11%, 16.67%, and 23.92% for A1, A2, and CK, respectively. The results suggest that only the 0.5 mM SA treatment effectively slowed the degradation of chlorophyll at 0°C.

Compared to normal temperature, Chlorophyll content was reduced by various concentrations of SA treatments under low temperature stress at −4°C. In contrast, the addition of SA at −4°C did not yield significant differences between the treatments and the CK, except for the 1.5 mM SA treatment (A3). This indicates that all treatment groups, with the exception of the 1.5 mM SA treatment, were effective in mitigating chlorophyll degradation (S1 Fig in S1 File and Table 2).

### Effects of different concentrations of SA on osmoregulatory substances in blueberry leaves under low temperature stress

Under normal conditions, the supplementation of SA resulted in a slight increase in the content of SS, SP, and Pro in blueberry leaves, with the exception of A1. In contrast, under low-temperature conditions at 4°C with SA supplementation, the levels of SS, SP, and Pro were significantly lower in treatment A2 compared to the CK, while all other treatments exhibited higher levels than the control. Specifically, the SS content was elevated in the 4°C treatments relative to normal temperature conditions. Similarly, the SP content increased. Conversely, Pro levels decreased, although Pro levels in the A5 increased by 4.01% in the 4°C treatment compared to normal temperature.

Supplementation of SA at 0°C resulted in significantly higher levels of SS, SP, and Pro in the A1 compared to the CK. The SS content was greater than that of the CK in all treatments except for the A3, while the SP and Pro contents were lower than the control in the remaining treatment. Compared to normal temperature, the SS content increased and the SP content showed increases. Additionally, the Pro level in the CK, A1, A2, and A4 showing increases of 42.55%, 29.66%, 20.31%, and 8.24%, while decreases of 10.26% and 3.64% were observed in the A3 and A5.

**Fig 1:**
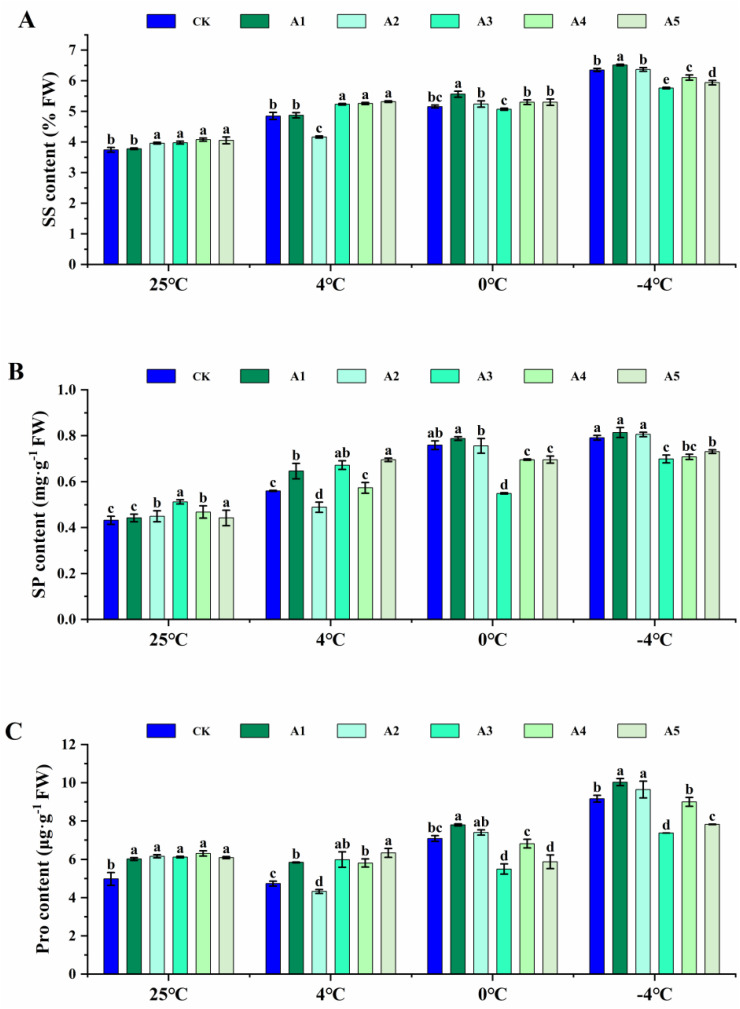
Effect of exogenous salicylic acid (SA) on osmotic adjustment substance in blueberry leaves under low temperature stress. (A) Soluble sugar (SS) content, (B) Soluble protein (SP) content, and (C) Free proline (Pro) content in blueberry leaves following exogenous supplementation with salicylic acid (SA) at concentrations of 0 (CK), 0.5 (A1), 1.0 (A2), 1.5 (A3), 2.0 (A4), and 2.5 (A5) mmol·L^-1^, assessed at normal temperature (25°C) and under low temperature stress (4°C, 0°C, and -4°C). Data are presented as mean ± SD (n=3). Significant difference analyses were conducted using one-way ANOVA, with different letters indicating statistically significant differences (LSD, *P* < 0.05).

Spraying SA under low-temperature at −4°C resulted in lower levels of SS, SP, and Pro compared to the CK across all treatments, with the exception of treatments A1 and A2. In comparison to room temperature, SS, SP, and Pro levels increased (Fig 1).

These results indicate that supplementation with varying concentrations of SA led to an increase in SS, SP, and Pro contents in blueberry leaves as the stress temperature decreased.

### Effect of different concentration SA on cell membrane system and ROS accumulation in blueberry leaves under low temperature stress

Exogenous SA significantly reduced the levels of REC, MDA, H_2_O_2_, and O_2_^‧-^ under normal temperature conditions. In contrast, under 4°C stress, the levels of REC, MDA, H_2_O_2_, and O_2_^‧-^ were markedly elevated compared to the treatments that included SA at normal temperature. Specifically, under 4°C stress, the addition of SA resulted in increases of 12.20%, 6.54%, 6.92%, and 12.54% in REC, MDA, H_2_O_2_, and O_2_^‧-^, respectively, in the treatment A2 when compared to the control, whereas the other treatments exhibited significantly lower levels.

The levels of REC, MDA, H_2_O_2_, and O_2_^‧-^ under 0°C stress were significantly higher than those observed after SA supplementation at room temperature. In the treatments A1 and A2, the levels of REC, MDA, H_2_O_2_, and O_2_^‧-^ were significantly lower than those in the CK when SA was applied at 0°C under low-temperature stress. Conversely, the levels of REC, MDA, H_2_O_2_, and O_2_^‧-^ were higher in the A3, A4, and A5 compared to the control.

**Fig 2:**
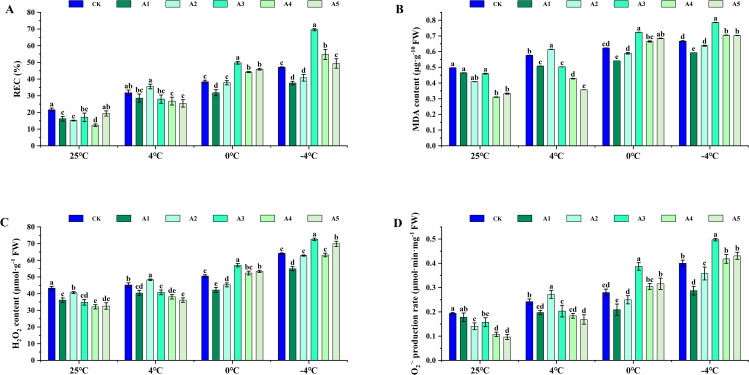
Effect of SA on cell membrane stability and reactive oxygen species (ROS) levels of blueberry leaves under low temperature stress. (A) Relative electrical conductivity (REC), (B) MDA content, (C) H_2_O_2_ content, and (D) O_2_.^-^ level in blueberry leaves following exogenous supplementation with SA at concentrations of 0 (CK), 0.5 (A1), 1.0 (A2), 1.5 (A3), 2.0 (A4), and 2.5 (A5) mmol·L^-1^, assessed at normal temperature (25°C) and under low temperature stress (4°C, 0°C, and -4°C). Data are presented as mean ± SD (n=3). Significant difference analyses were conducted using one-way ANOVA, with different letters indicating statistically significant differences (LSD, *P* < 0.05).

After spraying SA, the contents of REC, MDA, H_2_O_2_, and O_2_^‧-^ gradually increased with the intensification of low-temperature stress. Under low-temperature stress at −4°C, the levels of REC, MDA, H_2_O_2_, and O_2_^‧-^ in the treatments A1 and A2 were significantly lower than those in the CK following SA application. In contrast, the REC, MDA, H_2_O_2_, and O_2_^‧-^ levels in the treatments A3, A4, and A5 were higher compared to the CK treatment group, with the 1.5 mM SA treatment exhibiting the most pronounced increase in these parameters (Fig 2).

### Effect of different concentration SA on antioxidant enzyme activity of blueberry leaves under low temperature stress

**Fig 3:**
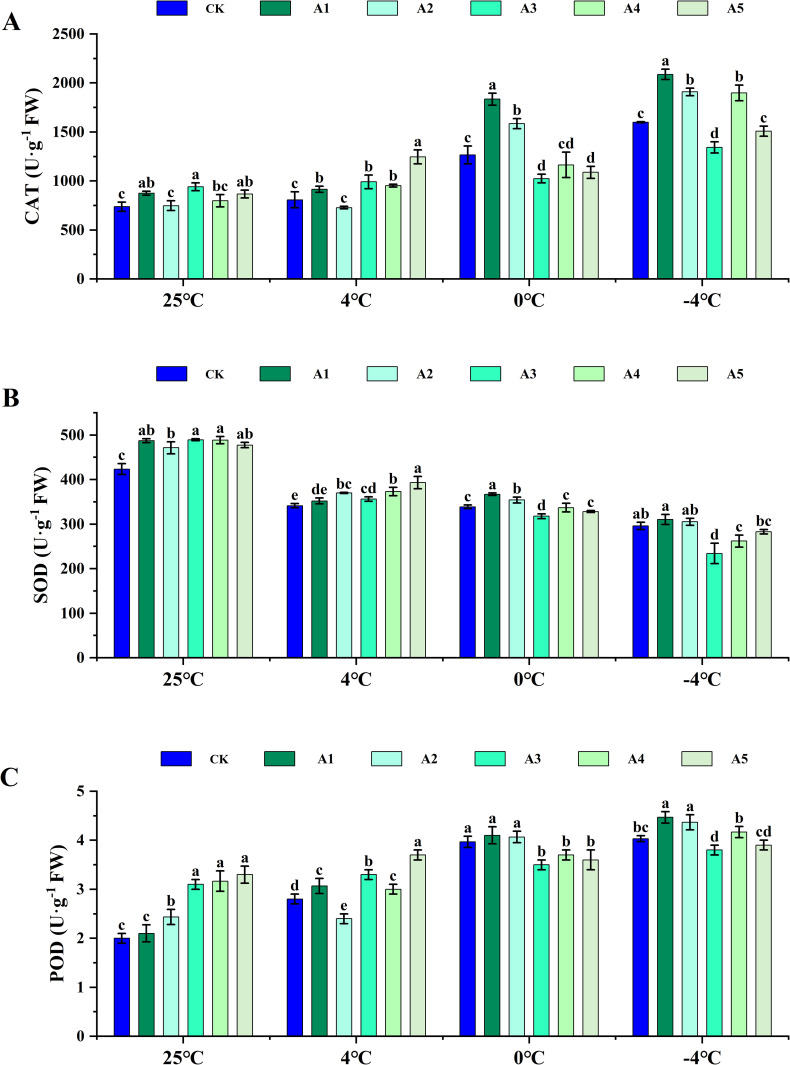
Regulatory effects of exogenous SA on antioxidant enzyme systems in blueberry leaves under low temperature stress. (A) Catalase (CAT) activity, (B) Superoxide dismutase (SOD) activity, and (C) Peroxidase (POD) activity in blueberry leaves following exogenous supplementation with SA at concentrations of 0 (CK), 0.5 (A1), 1.0 (A2), 1.5 (A3), 2.0 (A4), and 2.5 (A5) mmol·L^-1^, assessed at normal temperature (25°C) and under low temperature stress (4°C, 0°C, and -4°C). Data are presented as mean ± SD (n=3). Significant difference analyses were conducted using one-way ANOVA, with different letters indicating statistically significant differences (LSD, *P* < 0.05).

Under normal conditions, the activities of CAT, SOD, and POD in blueberry leaves were significantly higher after spraying with SA compared to the control. The CAT and POD activities in treatments A1, A3, A4, and A5 were greater than those in CK when sprayed with SA under low temperature stress at 4°C. Conversely, the CAT and POD activities in treatment A2 were significantly lower than those in the control by 9.90% and 14.29%, respectively. With the intensification of low temperature stress, the activities of CAT and POD gradually increased. Notably, under low temperature stress conditions at 4°C following SA supplementation, CAT and POD activities were lower than those in the control. However, after SA supplementation under 0°C and −4°C stress, CAT and POD activities were higher than the control in treatments A1 and A2, while they were lower than the control in treatments A3, A4, and A5 (Fig 3A, C).

The SOD activity of all treatments was higher than that of the control under low-temperature stress at −4°C, with increases of 3.14%, 8.44%, 4.34%, 9.27%, and 15.17%, respectively. However, as low-temperature stress intensified, SOD activity decreased significantly, particularly under −4°C stress. When spraying SA under low-temperature stress conditions at 0°C and −4°C, the SOD activities of treatments A1 and A2 were higher than that of the control, with A1 showing the most significant increase. In contrast, the SOD activities of the other SA treatments were lower than that of the control.

### Effects of different concentrations of SA on non-enzymatic responses of blueberry leaves under low temperature stress

**Fig 4:**
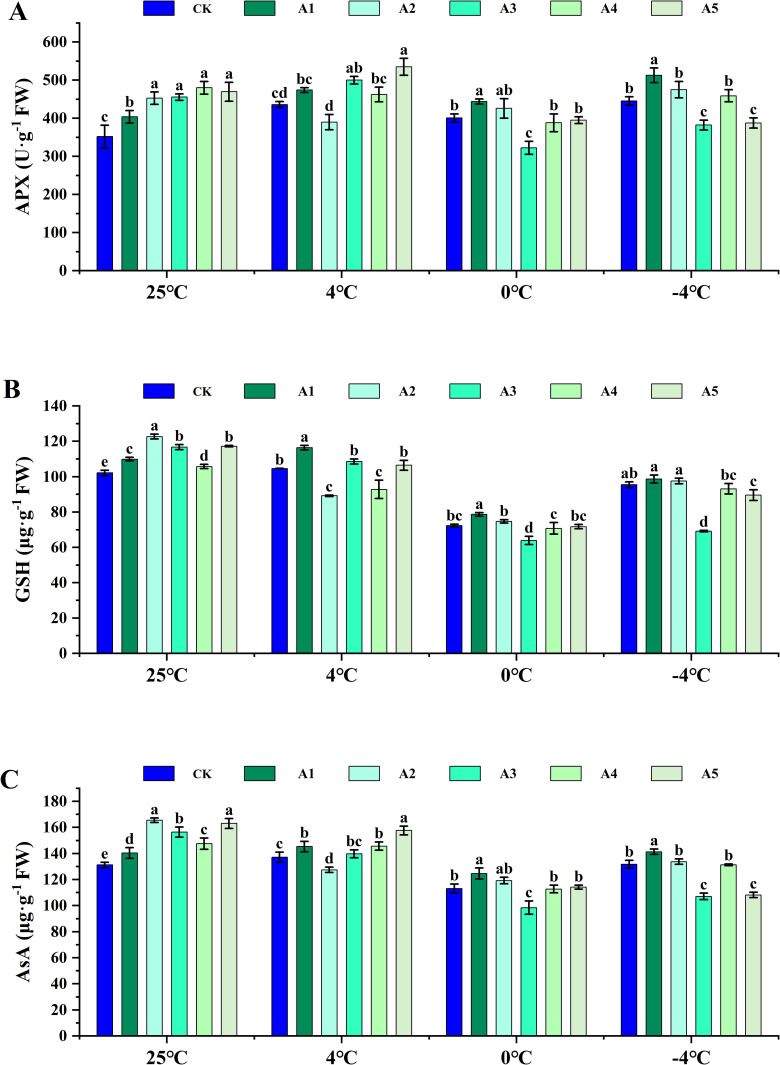
Effect of SA on the AsA-GSH antioxidant system of blueberry leaves under low temperature stress. (A) Ascorbate peroxidase (APX) activity, (B) Glutathione (GSH) content, and (C) Ascorbic acid (AsA) content in blueberry leaves following exogenous supplementation with salicylic acid (SA) at concentrations of 0 (CK), 0.5 (A1), 1.0 (A2), 1.5 (A3), 2.0 (A4), and 2.5 (A5) mmol·L^-1^, assessed at normal temperature (25°C) and under low temperature stress (4°C, 0°C, and -4°C). Data are presented as mean ± SD (n=3). Significant difference analyses were conducted using one-way ANOVA, with different letters indicating statistically significant differences (LSD, *P* < 0.05).

Under nornal conditions, the activity of APX increased (Fig 4A), and the contents of GSH and AsA were elevated following SA supplementation (Fig 4B, C). However, under low-temperature stress at 4°C and 0°C, APX activity was significantly inhibited, and the contents of GSH and AsA gradually decreased. After SA supplementation, both APX activity and GSH and AsA contents increased under 4°C stress compared to the CK. Nonetheless, this phenomenon varied with different SA concentration treatments. Specifically, APX activity decreased by 10.55% after A2 treatment compared to the control, while GSH content decreased by 14.77% and 11.29% under A2 and A4 treatments, respectively, compared to CK. Additionally, AsA content decreased by 7.07% after A2 treatment compared to CK.

Under low-temperature stress conditions at 0°C, the APX activity in the A1 and A2 treatment was significantly higher than that of the CK when sprayed with SA. Furthermore, the GSH content in the A1 and A2 treatments exceeded that of CK, with increases of 8.68% and 3.20%, respectively. Similarly, the AsA content in the A1 and A2 treatments was also greater than that of CK, with respective increases of 10.18%, 5.42%, and 0.87%.

Supplementation of SA under low-temperature stress at 4°C resulted in significantly higher activity of APX as well as increased levels of GSH and AsA in the A1 treatment compared to the control. Conversely, high concentrations of SA led to a reduction in APX activity, GSH, and AsA contents (Fig 4). In conclusion, a concentration of 0.5 mM SA positively influenced the non-enzymatic response mechanisms of blueberry leaves under low-temperature stress. However, as the concentration of supplemental SA increased, it appeared to exacerbate the effects of low-temperature stress on blueberries.

### PCA of relevant physiological indexes under different low temperature treatments

According to the results of the PCA, under the 4°C temperature treatment, PCA1 accounted for 73.815% of the variance, PCA2 accounted for 11.425%, resulting in a total variance explained of 85.240%. In the case of the 0°C temperature treatment, PCA1 explained 83.882% of the variance, PCA2 explained 5.085%, leading to a total variance explained of 88.967%. For the −4°C temperature treatment, PCA1 accounted for 81.052% of the variance, PCA2 accounted for 6.733%, with a total variance explained of 87.785%.

**Fig 5:**
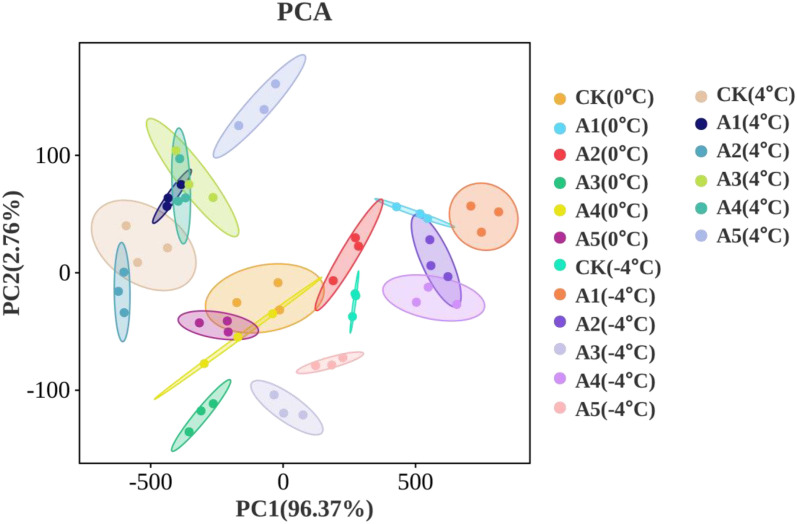
Principal component analysis (PCA) biplot of samples under different SA concentration treatments. Symbols represent replicate samples: CK (control), A1-A5 (treatment groups); colors indicate temperature conditions (0°C, 4°C,–4°C). PC1 explains 96.37 % of the total variance. The plot displays the clustering patterns of samples, illustrating the effect of temperature and treatment on the overall metabolic profile.

The elevated loadings of SS, SP, Pro, POD, CAT, APX, AsA, and GSH in PCA1 under low-temperature stress suggest their greater contribution to the first principal component across varying temperatures. Conversely, the diminished loadings of REC, MDA, O_2_^‧-^, and H_2_O_2_ in PCA1 imply their lesser impact on the first principal component. Furthermore, at 4 °C and 0°C, higher loadings of SOD in PCA2 indicate its increased contribution to the second principal component. Conversely, at −4°C, the negative loading of SOD in PCA2 suggests a divergent contribution compared to other temperature treatments. Notably, at 0°C, the relatively high loading of H_2_O_2_ in PCA2 signifies its greater contribution to the second principal component ( Fig 5 and S1 Table in S1 File). In summary, the PCA analysis reveals that variations in physiological indices under different temperature treatments reflect the significant impacts of distinct low-temperature conditions.

### Expression analysis of cold tolerance-related genes in blueberry after low temperature stress by different concentrations of SA

**Fig 6:**
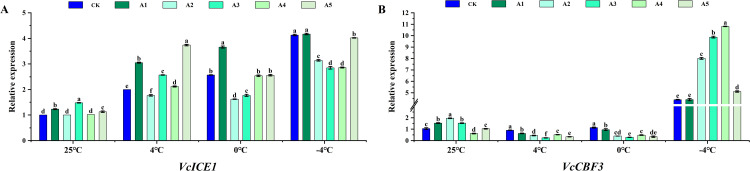
Effect of exogenous SA on the expression abundance of *VcICE1* and *VcCBF3* in blueberry leaves under low temperature stress. The relative expression of *VcICE1* (A) and *VcCBF3* (B) in blueberry leaves following exogenous supplementation with salicylic acid (SA) at concentrations of 0 (CK), 0.5 (A1), 1.0 (A2), 1.5 (A3), 2.0 (A4), and 2.5 (A5) mmol·L^-1^, assessed at normal temperature (25°C) and under low temperature stress (4°C, 0°C, and -4°C). Data are presented as mean ± SD (n=3). Significant difference analyses were conducted using one-way ANOVA, with different letters indicating statistically significant differences (LSD, *P* < 0.05).

To investigate the effects of SA treatment on low-temperature stress in blueberries, we assessed the transcript abundance of two genes, *VcICE1* and *VcCBF3* (Fig 6). Under low-temperature stress, the expression level of *VcICE1* increased as the temperature decreased. Different concentrations of SA exhibited varying effects on the expression abundance of *VcICE1* in blueberries; the most significant elevation in *VcICE1* expression was observed with 0.5 mM SA treatment. In contrast, *VcCBF3* showed no significant changes under 4°C and 0°C stress but exhibited a significant increase under −4°C stress, with the most pronounced rise occurring with 1–2 mM SA treatment. These results indicate that SA treatment enhances the low-temperature tolerance of blueberries.

As temperature decreases, the *VcICE1* exhibits a positive correlation with antioxidant-related indicators (e.g., SOD, CAT, APX activity, AsA, GSH content) and a negative correlation with REC, O_2_^‧-^, MDA, and H_2_O_2_ content (S2-S4 Figs in S1 File). Conversely, the expression level of *VcCBF3* significantly increases only under −4°C stress, with a low correlation observed with relevant physiological indicators. The variations in the correlation patterns of *VcICE1* and *VcCBF3* with physiological indicators following exogenous SA supplementation under different low-temperature treatments underscore their distinct responses to varying low-temperature stresses.

## Discussion

Low temperatures significantly restrict plant growth and development [28]. This environmental stress impairs both the physiology and morphology of plants. Morphologically, low temperatures cause yellowing, wilting, growth retardation, and potentially plant death [29]. Physiologically, low temperatures disrupt the plant cell membrane system, resulting in increased membrane permeability and accumulation of osmoregulatory substances [30]. This disturbance affects the stability of the intracellular environment and material exchange. Furthermore, low temperatures reduce photosynthesis, chlorophyll content, slow down metabolism, and increase the production of ROS, leading to oxidative damage [31]. Additionally, low temperatures hinder the uptake of minerals and nutrients by plants, further impacting plant growth and fruit quality [32].

Low concentrations of SA can confer beneficial physiological effects, including enhanced cold tolerance, disease resistance, and salt stress adaptation [33]. However, excessive external application or intracellular accumulation of SA may lead to phytotoxicity, manifesting as root damage, leaf scorching, and growth inhibition [34]. Consequently, in practical agricultural applications, exogenous SA concentrations should be minimized to balance efficacy and safety.

### Effect of exogenous SA on osmoregulatory substances

The findings indicated that exogenous SA can improve plant cold resistance by elevating the levels of osmotic regulators within cells. Proline serves as the primary osmotic regulator in plant cytoplasm, managing osmotic pressure and antioxidative processes effectively, thereby preserving cytoplasmic equilibrium with the external environment. Soluble sugars increase cellular osmotic concentration, ensuring rapid energy supply and membrane protection to maintain membrane protein stability. Soluble proteins boost cell water retention capacity, sustaining metabolic stability and long-term protection. These three components, centered around osmotic regulation, enhance plant resilience to stress through metabolic interactions, functional synergy, and resource allocation [35].

In this study, the osmotic regulating substances in blueberry leaves significantly increased under low-temperature stress in the pot experiment. Following SA supplementation, the levels of SS, SP, and Pro in blueberry leaves showed varying degrees of increase compared to the control. Specifically, under 4°C stress, the application of high concentrations of 2.5 mM SA resulted in the greatest enhancement in SS, SP, and Pro contents. Conversely, treatment with 1.5 mM SA exhibited a negative impact on the cell osmotic regulation system under 4°C stress. A concentration of 0.5 mM SA demonstrated superior maintenance of cell osmotic regulation balance under low-temperature stress. Conversely, when the SA treatment concentration exceeded 1.5 mM, the damage to cinnamon leaves intensified under low-temperature stress (Fig 1). In grape leaves, spraying 1 and 2 mM effectively increased the SS content and reduced damage to the vines caused by low temperatures. These findings suggest that there may be inconsistent responses to exogenous SA due to interspecies variations [24].

### Effects of exogenous SA on cell membrane systems

Cell membrane permeability is a crucial parameter in assessing plant cold tolerance [36]. Low-temperature stress disrupts the balance of oxidative metabolism in plant cells, leading to the overaccumulation of ROS [37]. This excess ROS triggers lipid peroxidation in the cell membrane, causing damage to the membrane system, a decline in its selective permeability, leakage of cellular contents, and ultimately an elevation in electrolyte leakage rates [38]. The accumulation of free radicals induces lipid peroxidation, resulting in the excessive production of MDA [39]. Research indicates that the exogenous application of SA can mitigate MDA levels in cucumber seedling leaves, enhancing the cold tolerance of cucumber seedlings [40].

In this study, it revealed a significant increase in the REC and MDA contents of blueberry leaves under low temperature stress in the pot experiment. However, supplementation with exogenous SA led to a notable reduction in REC and MDA contents compared to the control (Fig 2A, B). It is worth noting that the effective concentrations of SA varied depending on the degree of low temperature stress. Specifically, at 4°C stress, a high concentration of 2.5 mM SA demonstrated superior efficacy in safeguarding the cell membrane system. Conversely, under low temperatures of 0°C and −4°C, a lower concentration of 0.5 mM SA was more effective in preserving the cell membrane system, with a high concentration of SA exacerbating damage to the cell membrane system of blueberry leaves. Similarly, in Hami melons, the application of low concentrations of SA enhanced cold tolerance by modulating the SA response. Conversely, high concentrations of SA treatments had a detrimental impact on Hami melons [33].

### Effects of exogenous SA on the antioxidant system

Under low temperature stress, plants experience a disturbance in the dynamic equilibrium of ROS [41]. The resulting excessive accumulation of ROS leads to significant oxidative damage and cellular injury, ultimately inducing oxidative stress and impeding normal growth and development [42]. To counteract this, plants mitigate the surplus ROS by activating antioxidant enzyme systems such as SOD, POD, CAT, and APX, as well as utilizing ascorbic acid and the nonenzymatic antioxidant glutathione, GSH [43,44]. Numerous studies have illustrated that SA can enhance cold tolerance in plants by bolstering both enzymatic and nonenzymatic defense mechanisms [11,45].

Under low temperature stress, the H_2_O_2_ content and O_2_^‧-^ generating rate of the leaves increased significantly in the pot experiment (Fig 2C, D), while CAT and POD enzyme activities increased. Conversely, SOD enzyme activities decreased with decreasing stress temperature (Fig 3). Application of SA at normal temperature enhanced the antioxidant enzyme activity, AsA and GSH content of the leaves (Fig 4), thereby reducing H_2_O_2_ content and O_2_^‧-^ generating rate. Specifically, under 4°C stress, treatment with 2.5 mM SA had the most significant impact on CAT, POD, SOD, and APX activities. Under 0°C and −4°C stress, 0.5 mM SA treatment decreased H_2_O_2_ content and O_2_^‧-^ generating rate compared to controls, while enhancing antioxidant enzyme and GSH activity. Moreover, treatment with 1.0 mM exogenous SA notably boosted the antioxidant enzyme activity of watermelon. However, higher concentrations of exogenous SA diminished antioxidant enzyme activity, consequently reducing the cold resistance of watermelon [46].

PCA demonstrated that protective metabolites (SS, SP, etc.) primarily drove PCA1 under cold stress, while oxidative damage markers (REC, MDA, etc.) showed minimal contribution. Notably, SOD exhibited temperature-dependent loadings in PCA2 (positive at 4°C/0°C, negative at −4°C), with H_2_O_2_ specifically enhancing PCA2 at 0°C, revealing distinct temperature-regulated defense mechanisms (Fig 5 and S1 Table in S1 File).

### Effect of exogenous SA on the expression of cold-responsive genes

The ICE1-CBF-COR cold response pathway is a crucial signal transduction mechanism in plants’ response to cold stress [47,48]. In *Arabidopsis thaliana*, *AtICE1* binds to the MYC binding site on the *AtCBF3* promoter, leading to the activation of *AtCBF3* expression. Consequently, *AtCBF3* stimulates *AtCOR* transcription, thereby bolstering cold tolerance in *Arabidopsis thaliana* [49,50]. The CBF transcription factor plays a significant role in blueberry response to cold stress [25,51]. Research findings indicate that SA can ameliorate organ freezing injury and enhance cold tolerance in peach under cold conditions, with a notable increase in *CBF* expression post-SA treatment [52]. SA mitigates chilling injury in grapevines by upregulating the expression of *CBF1*, *CBF2*, and *CBF3* under chilling stress. In tobacco leaves, *CbICE53*, akin to *Arabidopsis AtICE1*, enhances plant tolerance to low temperatures by activating downstream cold response pathways [53,54]. The qRT-PCR analysis revealed a significant upregulation of *VcICE1* expression under low temperature stress following SA treatment compared to the control (Fig 6). Conversely, the expression level of *VcCBF3* did not show a significant alteration under low temperature stress above 0°C, but exhibited a notable increase under −4°C stress conditions. These findings suggest that *VcICE1* and *VcCBF3* are responsive to low temperatures, particularly in the presence of exogenous SA.

## Conclusions

This study has demonstrated that varying concentrations of SA can significantly enhance the cold tolerance of blueberries; however, the effects differ depending on the concentration used. Under 4°C stress, higher concentrations of SA were found to mitigate low-temperature damage in blueberries by increasing the levels of osmoregulatory substances in the leaves, enhancing the activity of antioxidant system enzymes, and decreasing the accumulation of ROS. Notably, the treatment with 2.5 mM SA yielded the most favorable results. Conversely, under 0°C and −4°C stress, lower concentrations of SA proved to be more effective in improving cold tolerance, with the most pronounced difference observed at 0.5 mM SA compared to the control. Therefore, the pot experiment demonstrated that early spring application of 0.5 mM SA significantly enhanced the cold tolerance of blueberry plants under low-temperature stress. However, the practical applicability of this effect requires further validation through field trials.

## Supporting information

S1 File Supplemental materials. **This file includes S1 Fig-S4 Fig and S1 Table. S1 Fig.** Phenotypes of blueberry lines subjected to varying concentrations of salicylic acid (SA) under low-temperature stress at −4°C. **S2 Fig.** Relation analysis of various physiological indexes of blueberry leaves under low temperature stress at 4°C. **S3 Fig.** Relation analysis of various physiological indexes of blueberry leaves under low temperature stress at 0°C. **S4 Fig.** Relation analysis of various physiological indexes of blueberry leaves under low temperature stress at −4°C. **S1 Table.** PCA of various physiological indices of blueberry leaves under low temperature stress.
(ZIP)
