# Supplementary material for: Effect of exogenous salicylic acid spray on enhancing cold tolerance in ‘Northland’ blueberry plants under low-temperature stress
Source: PLoS One. 2025 Dec 10;20(12):e0338327. doi: 10.1371/journal.pone.0338327 (PMC12694834; doi:10.1371/journal.pone.0338327)
Supplement: S1 File — This file includes S1 Fig-S4 Fig and S1 Table. S1 Fig. Phenotypes of blueberry lines subjected to varying concentrations of salicylic acid (SA) under low-temperature stress at −4°C. S2 Fig. Relation analysis of various physiological indexes of blueberry leaves under low temperature stress at 4°C. S3 Fig. Relation analysis of various physiological indexes of blueberry leaves under low temperature stress at 0°C. S4 Fig. Relation analysis of various physiological indexes of blueberry leaves under low temperature stress at −4°C. S1 Table. PCA of various physiological indices of blueberry leaves under low temperature stress. (ZIP) [file pone.0338327.s001.zip › S1 Figure.docx]

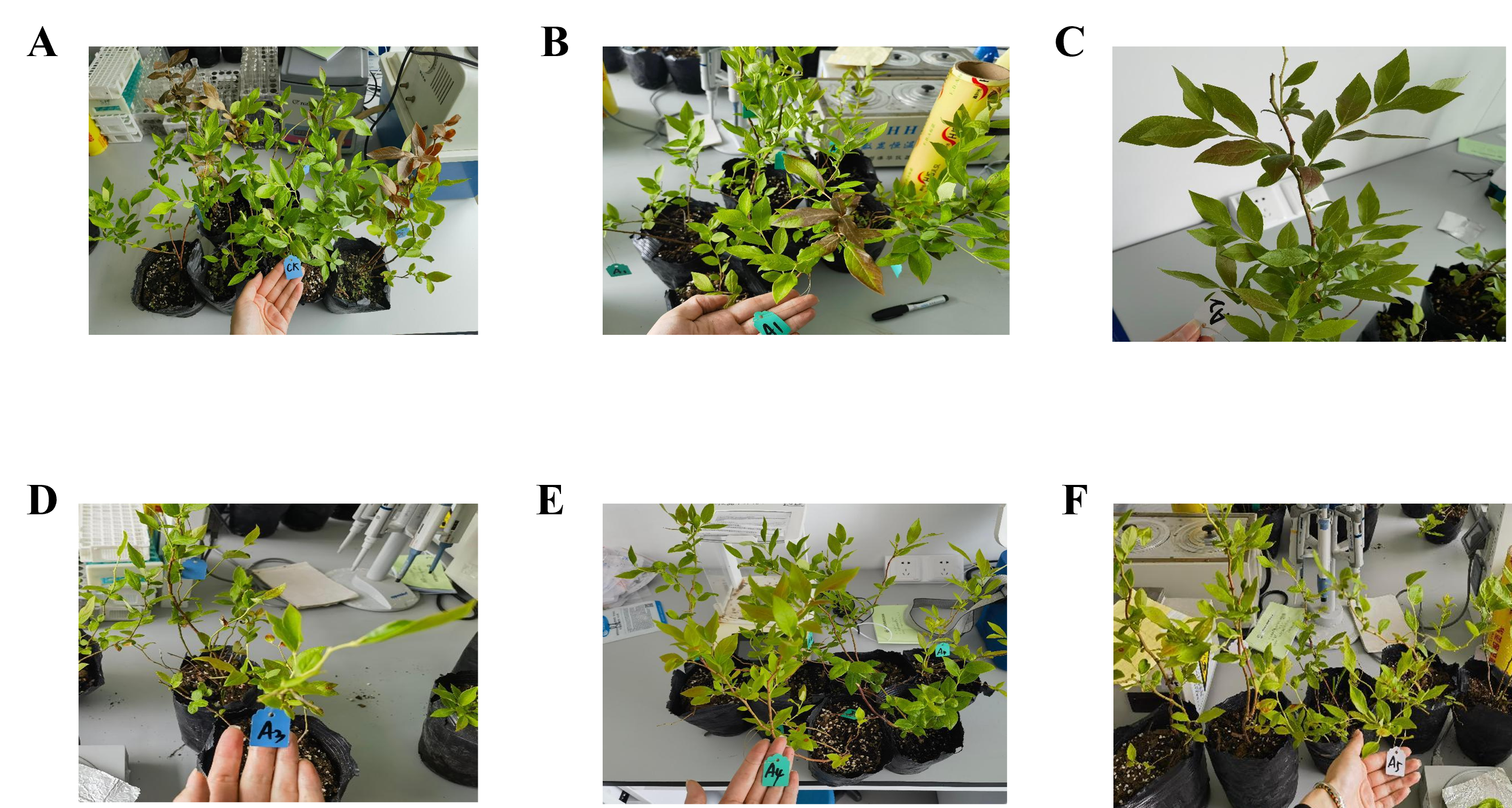


**S1 Figure.** Phenotypes of blueberry lines subjected to varying concentrations of salicylic acid (SA) under low-temperature stress at -4°C. The concentrations of supplemental SA are designated as follows: A, CK, 0 mmol‧L^-1^; B, A1, 0.5 mmol‧L^-1^；C, A2, 1.0 mmol‧L^-1^；D, A3, 1.5 mmol‧L^-1^；E, A4, 2.0 mmol‧L^-1^；F, A5, 2.5 mmol‧L^-1^. Observations were conducted after a 16-h treatment period under low-temperature conditions.
