# Supplementary material for: Effect of exogenous salicylic acid spray on enhancing cold tolerance in ‘Northland’ blueberry plants under low-temperature stress
Source: PLoS One. 2025 Dec 10;20(12):e0338327. doi: 10.1371/journal.pone.0338327 (PMC12694834; doi:10.1371/journal.pone.0338327)
Supplement: S1 File — This file includes S1 Fig-S4 Fig and S1 Table. S1 Fig. Phenotypes of blueberry lines subjected to varying concentrations of salicylic acid (SA) under low-temperature stress at −4°C. S2 Fig. Relation analysis of various physiological indexes of blueberry leaves under low temperature stress at 4°C. S3 Fig. Relation analysis of various physiological indexes of blueberry leaves under low temperature stress at 0°C. S4 Fig. Relation analysis of various physiological indexes of blueberry leaves under low temperature stress at −4°C. S1 Table. PCA of various physiological indices of blueberry leaves under low temperature stress. (ZIP) [file pone.0338327.s001.zip › S1 Table.docx]

**S1 Table. PCA of various physiological indices of blueberry leaves under low temperature stress.**

| Treatment Temperature | Physiological indicators | Load factor | | Commonality  (common factor variance) |
| --- | --- | --- | --- | --- |
|  |  | PCA1 | PCA2 |  |
| 4℃ | SS | 0.88 | 0.107 | 0.843 |
|  | SP | 0.892 | 0.27 | 0.936 |
|  | Pro | 0.952 | 0.062 | 0.911 |
|  | REC | -0.856 | -0.052 | 0.744 |
|  | MDA | -0.937 | 0.26 | 0.977 |
|  | O_2_^·-^ | -0.924 | 0.009 | 0.897 |
|  | H_2_O_2_ | -0.938 | 0.064 | 0.905 |
|  | SOD | 0.473 | -0.808 | 0.88 |
|  | POD | 0.951 | 0.068 | 0.927 |
|  | CAT | 0.929 | -0.135 | 0.897 |
|  | APX | 0.94 | 0.087 | 0.91 |
|  | AsA | 0.923 | 0.015 | 0.889 |
|  | GSH | 0.5 | 0.792 | 0.954 |
|  | CHl | 0.709 | -0.038 | 0.743 |
| 0℃ | SS | 0.728 | 0.495 | 0.774 |
|  | SP | 0.919 | -0.03 | 0.845 |
|  | Pro | 0.93 | -0.138 | 0.883 |
|  | REC | -0.973 | 0.036 | 0.947 |
|  | MDA | -0.924 | 0.073 | 0.86 |
|  | O_2_^·-^ | -0.959 | 0.004 | 0.92 |
|  | H_2_O_2_ | -0.961 | 0.149 | 0.945 |
|  | SOD | 0.936 | -0.099 | 0.886 |
|  | POD | 0.853 | -0.341 | 0.844 |
|  | CAT | 0.914 | -0.149 | 0.857 |
|  | APX | 0.904 | -0.034 | 0.819 |
|  | AsA | 0.906 | 0.029 | 0.822 |
|  | GSH | 0.936 | 0.024 | 0.877 |
|  | CHl | 0.922 | -0.105 | 0.86 |
| -4℃ | SS | 0.955 | -0.014 | 0.912 |
|  | SP | 0.868 | -0.257 | 0.82 |
|  | Pro | 0.952 | 0.217 | 0.953 |
|  | REC | -0.934 | 0.201 | 0.914 |
|  | MDA | -0.966 | 0.093 | 0.941 |
|  | O_2_^·-^ | -0.96 | 0.002 | 0.922 |
|  | H_2_O_2_ | -0.933 | -0.192 | 0.907 |
|  | SOD | 0.859 | -0.322 | 0.841 |
|  | POD | 0.863 | 0.323 | 0.849 |
|  | CAT | 0.867 | 0.425 | 0.932 |
|  | APX | 0.898 | 0.326 | 0.912 |
|  | AsA | 0.899 | 0.309 | 0.905 |
|  | GSH | 0.903 | -0.041 | 0.816 |
|  | CHl | 0.664 | 0.132 | 0.459 |
